# Mitochondrial Dysfunction in Acute Kidney Injury: Intersections Between Chemotherapy and Novel Cancer Immunotherapies

**DOI:** 10.3390/biom16010120

**Published:** 2026-01-12

**Authors:** Zaroon Zaroon, Carlotta D’Ambrosio, Filomena de Nigris

**Affiliations:** Department of Precision Medicine, University of Campania “Luigi Vanivtelli”, 80138 Naples, Italy; zaroon.zaroon@unicampania.it (Z.Z.);

**Keywords:** acute kidney injury (AKI), mitochondrial dysfunction, immune checkpoint inhibitor, inflammasome, nephrotoxicity

## Abstract

Acute kidney injury (AKI) remains a major clinical challenge, with high morbidity and limited therapeutic options. In recent years, mitochondria have gained considerable attention as key regulators of the metabolic and immune responses during renal injury. Beyond their classical role in ATP production, mitochondria participate directly in inflammatory signaling, releasing mitochondrial DNA and other DAMPs that activate pathways such as TLR9, cGAS–STING, and the NLRP3 inflammasome. At the same time, immune cells recruited to the kidney undergo significant metabolic shifts that influence whether injury progresses or resolves. Increasing evidence also shows that immune-modulating therapies, including immune checkpoint inhibitors and innovative cell-based immunotherapies, can influence mitochondrial integrity, thereby altering renal susceptibility to injury. This review first summarizes the established knowledge on mitochondrial dysfunction in AKI, with emphasis on distinct mechanistic pathways activated by chemotherapy and immunotherapy. It then discusses emerging mitochondrial-targeted therapeutic strategies, logically integrating preclinical insights with data from ongoing and proposed clinical trials to present a coherent translational outlook.

## 1. Introduction

Acute kidney injury (AKI) can be defined as the rapid decrease in glomerular filtration rate (GFR) causing the dysregulation of fluid, electrolyte, acid-base balance; as well, it also leads to the accumulation of nitrogenous waste products [1]. Clinically, AKI ranges from subtle and transient changes detectable only through biomarkers to overt anuric renal failure requiring urgent intervention [2,3]. It has been understood that AKI is an independent risk factor of the onset and development of end-stage kidney disease and chronic kidney disease (CKD) [4]. Many years of research have not yet been translated into an effective drug treatment that can repair cellular damage. It demonstrates that we are direly in need of knowing what causes AKI and how we can go about specific pathways [5]. Diagnosis currently relies on the Kidney Disease: Improving Global Outcomes (KDIGO) criteria, which define AKI based on changes in serum creatinine or urine output over relatively short time intervals [1]. While widely adopted, these indicators are indirect and often detect injury only after substantial cellular stress has occurred. For this reason, several early biomarkers, including neutrophil gelatinase-associated lipocalin (NGAL), kidney injury molecule-1 (KIM-1), and the cell cycle arrest markers TIMP-2·IGFBP7, are being explored, particularly in high-risk contexts such as cardiothoracic surgery or exposure to nephrotoxic anticancer therapies [5,6].

**Figure 1:**
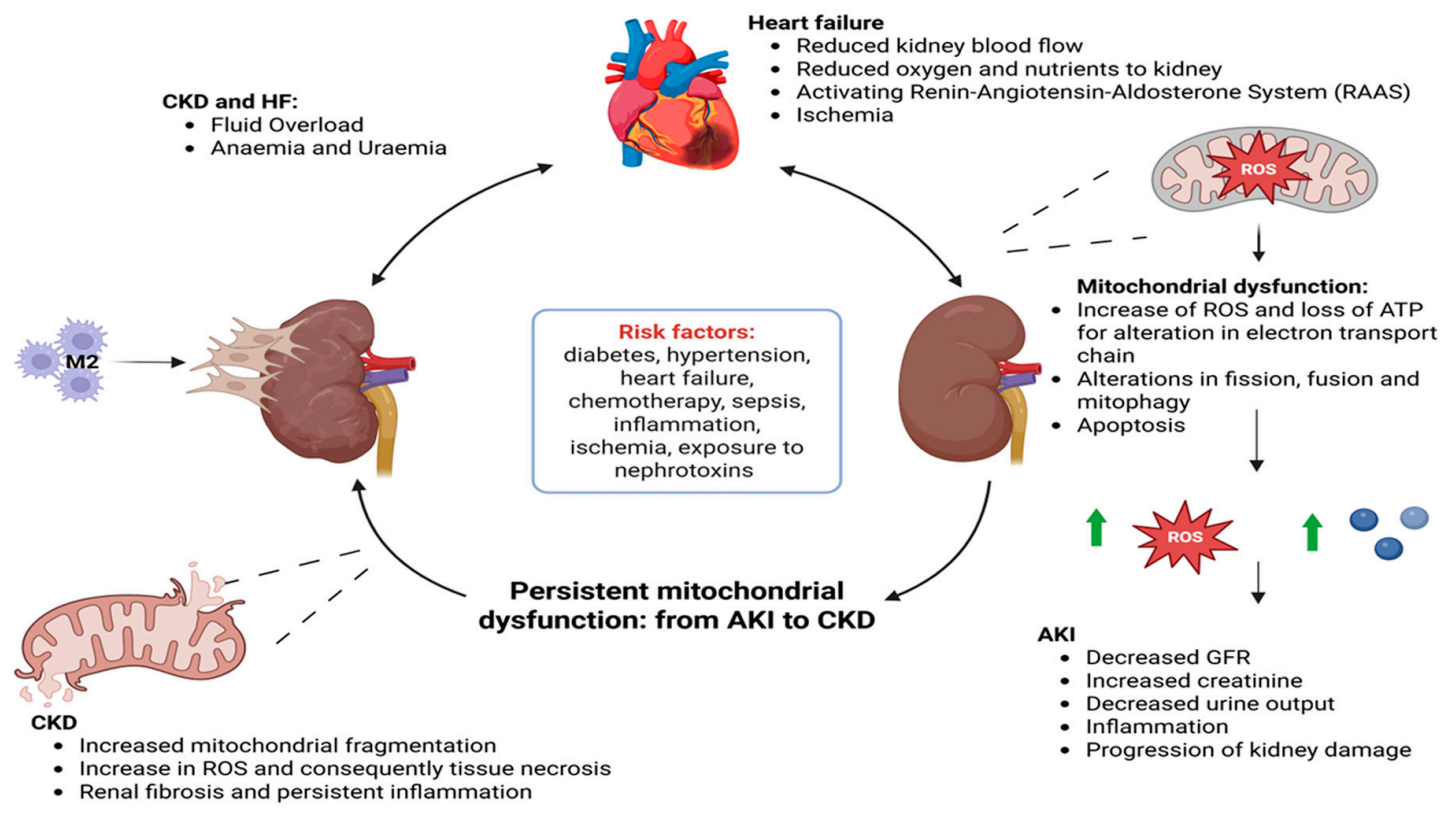
Interplay between cardiovascular disease, systemic inflammation, and kidney injury: the mitochondrial nexus. Chronic heart disease and heart failure can promote both acute kidney injury (AKI) and progression to chronic kidney disease (CKD) and vice versa; while AKI is characterized by acute, potentially reversible, mitochondrial dysfunction, CKD is associated with persistent mitochondrial damage, metabolic reprogramming, and maladaptive repair. Massive release of pro-inflammatory cytokines, including IL-6, IL-1Β, IFN-Γ, and GM-CSF, by infused CAR T cells and activated host immune cells drives a systemic hyperinflammatory state.

A common feature in almost all causes of AKI is damage to the renal tubular epithelium, particularly the proximal tubule. These cells have very high energy needs because they reabsorb most of the filtered water, electrolytes, and solutes [7]. They meet this demand largely through mitochondrial oxidative phosphorylation, relying heavily on fatty-acid oxidation for ATP production [8]. As a result, proximal tubular cells have an unusually dense and specialized network of mitochondria. This arrangement allows for efficient solute transport under normal conditions but also makes the tubule highly sensitive to mitochondrial damage from ischemia, toxins, or systemic inflammation. Recent studies have changed how we view mitochondria in AKI. Rather than being just passive victims of injury, mitochondria are now seen as central regulators of cell survival. In addition to producing ATP, they control redox balance, calcium homeostasis, and both pro-survival and pro-death signaling [9]. When mitochondria fail, a self-amplifying cycle begins: impaired electron transport lowers ATP, increases reactive oxygen species (ROS), and triggers pathological mitochondrial fragmentation through Drp1-mediated fission [10,11]. If quality-control mechanisms like mitophagy are overwhelmed, damaged mitochondria accumulate, eventually causing outer-membrane permeabilization, the release of pro-apoptotic factors, and cell death [12]. AKI commonly occurs in the setting of heart failure and chronic heart diseases, and it also represents a major complication of chemotherapy and immunotherapy used for the treatment of a wide range of malignancies. Within the cardiovascular setting, AKI often arises during ischemia–reperfusion events, including cardiac surgery, myocardial infarction, and cardiogenic shock, and is mechanistically associated with impaired mitochondrial function and the inhibition of mitophagy [13]. Chronic heart failure further predisposes the kidney to injury through persistent inflammation and altered perfusion, contributing to the progression to chronic kidney disease (CKD) [14] (Figure 1).

However, while AKI is characterized by acute, potentially reversible mitochondrial dysfunction, CKD is associated with persistent mitochondrial damage, metabolic reprogramming, and maladaptive repair.

Nephrotoxicity is a drawback of both contemporary immunotherapies and conventional chemotherapy in cancer patients. Cisplatin, for example, directly damages mitochondrial DNA and impairs oxidative phosphorylation [15]. New oncology therapy approaches increasingly call for cautious dose modifications depending on the renal function [16]. Mitochondrial integrity within the tubule can be compromised further by immune checkpoint inhibitors and CAR T-cell treatment, which can cause severe systemic inflammation or cytokine release syndromes. In cancer patients, the kidney is exposed to multiple nephrotoxic threats [17,18]. Across these clinical scenarios, mitochondrial health consistently emerges as a key determinant of renal vulnerability and recovery.

This review discusses mitochondrial metabolism and dysfunction in AKI, with emphasis on molecular mechanisms including impaired bioenergetics, oxidative stress, abnormal dynamics, and defective quality control that drive tubular injury across ischemic, chemotherapeutic, and immunotherapy-related contexts [19,20]. We also highlight translational opportunities such as mitochondrial-targeted therapies, NAD^+^ restoration, antioxidants, and next-generation tools, including kidney organoids and organ-on-chip models, which may help protect renal function in the clinical settings where patients are most vulnerable.

## 2. Mitochondrial Vulnerability in AKI

The kidney is highly metabolically active, and its large energy requirements make it particularly vulnerable to acute injury. Much of this vulnerability stems from the renal tubule, particularly the proximal tubule, which performs the bulk of solute and water reabsorption and depends heavily on mitochondrial energy production to do so. A detailed description of renal metabolism is reported in many reviews [21], but here, we focus on some key concepts that explain why mitochondria sit at the center of AKI pathophysiology.

Oxidative phosphorylation is the major energy source in proximal cells of the tubules. A minimal role is played by glycolysis. The breakdown of fatty acids occurs in the citric acid cycle and electron transport chain, which provide the majority of the ATP [22]. The system functions perfectly under normal conditions: the tubule is filled with long mitochondria, which align following the basolateral membrane, which is near the ATP-consuming Na^+^/K^+^-ATPase pumps. This tight coupling maximizes energy efficiency and supports the high ATP demand of tubular reabsorption [23,24,25].

The kidney has a high concentration of mitochondria per gram of tissue, mainly in the tubular cells in the proximal and thick ascending limb. The proximal convoluted tubule and the ascending loop of Henle are responsible for reabsorbing the glomerular filtration produced daily. This makes them the most energy demanding tissue in the body. The main driver behind this is the Na+/K+ ATPase pump, which consumes up to 70–80% of the cellular ATP to create the electrochemical gradient required for the transport of solutes such as glucose and amino acids [26]. To cope with extraordinary demand, proximal tubular cells of the kidney, particularly those in the S3 section, are the most mitochondria-enriched cells in the body. They are established to continue using oxidative phosphorylation, and they do not readily change to glycolysis when they are stressed [27,28]. The tubules are also highly vulnerable, as they depend on mitochondria to a large extent. Even little disruptions can lead to the rapid halt of energy production, and the tubules will be immediately dysfunctional, resulting in AKI. The production of ATP in these cells is due to the breakdown of free fatty acids (β-oxidation). The products integrate into the citric acid cycle and electron transport chain, where a large amount of ATP is generated out of a single molecule of substrate [25]. The proximal tubule is adjusted to these energy requirements by the structure of its cells. Electron microscopy reveals that the mitochondria are nearby, long, and aligned along the inner fold of the cell membrane Na^±^/K 3-ATPase pump. This will create micro-domains for energy transfer to support active transport. One of the initial processes in AKI is the disruption of the association between mitochondria and the membrane. Reduced mitochondrial function and ATP depletion impair Na^+^/K^+^-ATPase activity, leading to the rapid loss of cellular ion homeostasis [23,24]. Clinical results, along with in vitro and in vivo studies, have confirmed that mitochondrial damage in proximal tubule cells occurs before overt tubular necrosis and functional deterioration, indicating that mitochondrial dysfunction is a key cause of AKI [29,30].

## 3. Mechanism Involved in Mitochondrial Damage During AKI

**Figure 2:**
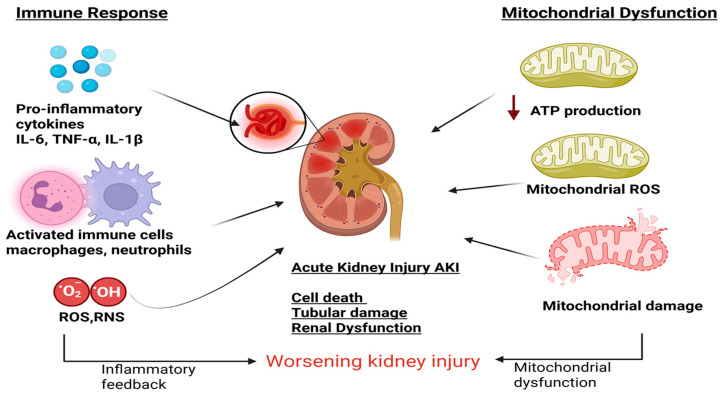
Bidirectional cross-talk between immune activation and mitochondrial dysfunction in AKI. Pro-inflammatory cytokines and activated immune cells promote oxidative stress, leading to mitochondrial damage, impaired ATP production, and increased mitochondrial ROS generation. Mitochondrial dysfunction further amplifies inflammation, creating a vicious cycle that accelerates tubular injury, cell death, and renal dysfunction.

In AKI, mitochondrial dysfunction acts both as an initiating insult and as a consequence of tubular cell injury, creating a self-amplifying feedback loop that accelerates disease progression. Tubular cells are highly metabolically active and particularly susceptible to energy deficits. Impaired mitochondrial bioenergetics reduces ATP production, disrupts ion transport, and generates excessive reactive oxygen species. This activates local inflammatory pathways, further damaging mitochondria and tubular structures (Figure 2), reinforcing injury and limiting renal recovery.

The maintenance of a functional mitochondrial network within renal tubular epithelial cells relies on an intricate and highly regulated quality control system encompassing mitochondrial biogenesis, dynamics, mitophagy, and signaling homeostasis. Dysregulation of these interdependent pathways has been consistently identified as a defining pathogenic feature of acute kidney injury (AKI) [15,31]. The PGC-1α/NRF1/TFAM axis regulates mitochondrial biogenesis by controlling the transcription of mitochondrial DNA and the formation of oxidative phosphorylation complexes. AKI severely lowers PGC-1α levels, which restricts the recovery of respiratory function and mitochondrial mass. Mitochondrial morphology depends on the balance between fusion and fission. MFN1, MFN2, and OPA1 promote fusion, whereas Drp1 drives fission. AKI consistently shifts this balance toward fragmentation through Drp1 activation and reduced OPA1 processing [32]. Fragmented mitochondria display lower ATP output and increased ROS generation, reinforcing injury mechanisms. Dysfunctional mitochondria are primarily removed via PINK1–Parkin-dependent mitophagy. In AKI, mitophagy can be either insufficient or excessively activated, depending on the severity and context of the insult [31]. In both cases, inadequate clearance of damaged mitochondria amplifies oxidative stress and lowers the threshold for apoptotic signaling. Loss of mitochondrial membrane integrity is a pivotal event. Opening of the mitochondrial permeability transition pore (mPTP) disrupts membrane potential, halts ATP generation, and facilitates the release of cytochrome-c and other pro-apoptotic mediators [33]. Caspase activation follows, particularly in ischemia–reperfusion and cisplatin-induced AKI.

**Figure 3:**
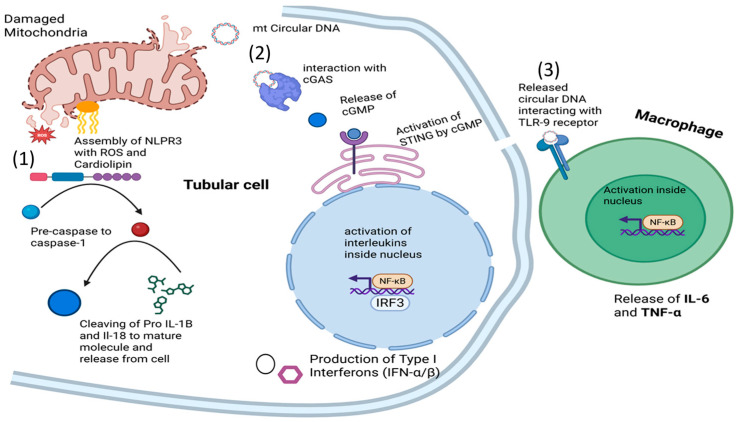
Mitochondria initiate sterile inflammation via mtDAMP signaling. (**1**) Mitochondrial ROS and cardiolipin activate the NLRP3 inflammasome, leading to maturation and release of IL-1β and IL-18. (**2**) Cytosolic mtDNA activates the cGAS–STING pathway for cytokine and interleukin production. (**3**) Extracellular mtDNA engages Toll-like receptors on immune cells.

Damaged mitochondria release mtDNA, formyl peptides, and other DAMPs that activate TLR9, NLRP3, and cGAS–STING signaling pathways. mtDNA-driven NLRP3 activation has been repeatedly associated with proximal tubular injury across several AKI models. Excess ROS production from the electron transport chain leads to lipid peroxidation, protein oxidation, and DNA damage. Oxidative stress further inhibits mitochondrial enzymes, reduces respiratory capacity, and perpetuates cellular injury. Together, these mechanisms outline a convergent pattern of mitochondrial impairment characterized by defective biogenesis, excessive fragmentation, impaired mitophagy, altered membrane permeability, inflammatory activation, and ROS accumulation that drives the initiation and progression of acute tubular damage [34]. (Figure 3).

## 4. Mitochondria as Immune Signaling Platforms During AKI

Mitochondria are potent activators of the innate immune system. Under stress conditions, such as ischemia, nephrotoxicity, or sepsis, mitochondrial damage triggers the release of mitochondrial DNA (mtDNA), cardiolipin, and N-formyl peptides into the cytosol and, in some cases, into the systemic circulation [35,36]. Extracellular mtDNA acts as a danger signal, engaging Toll-like receptor 9 (TLR9) on tubular epithelial cells and macrophages, thereby activating NF-κB signaling and promoting the release of IL-6 and TNF-α [36]. Cytosolic mtDNA can also activate the cGAS–STING pathway, leading to type I interferon responses and sustained inflammation [37]. Cardiolipin exposure on the outer mitochondrial membrane facilitates assembly of the NLRP3 inflammasome, resulting in caspase-1 activation and secretion of IL-1β and IL-18 [38]. These signaling cascades amplify local and systemic inflammation even in the absence of pathogens, defining the sterile inflammation characteristic of ischemic and toxic AKI. Mitochondrial ROS further oxidize mtDNA, enhancing its immunogenic potential and creating a self-perpetuating loop of oxidative and inflammatory injury. Inhibition of TLR9, STING, or NLRP3 has been described in preclinical research to reduce renal inflammation and maintain renal function [11,36,39]. The pyrin domain of NLR containing 3 (NLRP3) was highlighted in a study by Lin and colleagues that examined the pathogenic mechanism of contrast-induced acute kidney injury (CI-AKI). In a mouse model, it was described that suppression of the NLR3 inflammasome reduces apoptosis in AKI by upregulating hypoxia-inducible factor 1-alpha (HIF1A) and BCL2 interacting proteins 3 (BNIP3)-mediated mitophagy [26]. Mitochondrial dysfunction activates the NLRP3 inflammasome, contributing to renal disease, as supported by robust evidence from in vitro studies, animal models, and clinical investigations. Research in renal tubular epithelial cells has identified specific mitochondrial components that act as NLRP3 agonists. Direct mitochondrial injury, induced by complex I inhibitors (e.g., rotenone) or ATP synthase inhibitors (e.g., oligomycin), causes collapse of the mitochondrial membrane potential and a burst of mitochondrial reactive oxygen species (mtROS). These mtROS and released mitochondrial danger signals not only drive local tubular injury but can also enter the systemic circulation, activating immune cells and promoting cytokine release. This cascade may trigger widespread inflammation, endothelial dysfunction, and contribute to multi-organ injury beyond the kidney [40].

Apart from ROS, the release of mitochondrial DNA (mtDNA) into the cytosol is also a key event. Oxidized mtDNA generated under stress conditions directly binds to and activates the NLRP3 inflammasome [41]. Furthermore, the recruitment of NLRP3 to the mitochondria is facilitated by proteins like the mitochondrial antiviral signaling protein (MAVS) and mitofusin 2 (Mfn2), creating a physical platform for inflammasome oligomerization [42]. Recent work has shown that, upon stimulation, NLRP3 translocates to mitochondria-associated endoplasmic reticulum membranes (MAMs) and the dispersed trans-Golgi network (dTGN), a process regulated by mitochondrial-derived cardiolipin and protein kinase D (PKD) signaling [24,43]. Notably, the mitochondrial ROS scavenger MitoTEMPO effectively suppresses NLRP3 inflammasome assembly and prevents IL-1β secretion in these models, underscoring the central role of mtROS in driving this inflammatory response. Data from AKI mouse models support the significance of this mitochondrial-NLPR3 axis from a pathophysiological point. Peak NLPR3 activation and Il-1β maturation in ischemia–reperfusion injury models are preceded by cytosolic mtDNA and mitochondrial damage indicators [44,45]. Protection against IRI is given by the genetic deletion of NLPR3 and CASP1 in mice, which reduces tubular damage, inflammation, and consequent fibrosis. Significantly, this axis is disrupted by the therapies that improve mitophagy or restore mitochondrial integrity. For example, in rat IRI models, the mitochondria-targeted peptide SS-31 (Elamiptretide) decreases the mtROS, maintains cristae structure, and attenuates NLRP-3-driven inflammation and death [46]. In cisplatin-induced AKI, the PINK1/Parkin mitophagy pathway is activated. Its inhibition leads to increased pyroptosis and renal injury, whereas intact mitophagy restrains NLRP3 activation by removing damaged mitochondria and mitochondrial inflammatory triggers. Evidence from human studies supports the existence of this axis, although current clinical interventions targeting it remain largely ineffective. Nevertheless, there is growing potential for pharmacological agents that indirectly modulate this pathway [47,48]. In this context, Elamipretide has been shown to improve renal function and reduce tissue hypoxia in patients with atherosclerotic renal artery stenosis [49]. Future investigation in AKI is warranted, as the NLRP3 inhibitor dapansutrile has progressed to Phase II trials for inflammatory diseases [50], highlighting the translational relevance of this pathway. Additional human evidence of mtDNA and other mitochondrial mediators as triggers of inflammatory signaling is derived from SARS-CoV-2 infection. These patients showed increased urinary mtDNA levels associated with heightened cytokine production in peripheral blood mononuclear cells and activation of macrophage-related pathways [51]. This data suggests that immune activation in human AKI is primarily caused by mitochondrial damage and inflammasome formation, which exacerbates renal injury by inducing cytokine release and pyroptosis. Therefore, this established pathway constitutes a key therapeutic target for limiting inflammation in AKI. However, human data remain limited, often observational, and are derived from small cohorts, highlighting important translational gaps between preclinical models and clinical AKI.

## 5. Acute Kidney Injury and Mitochondrial Dysfunction in Cancer Chemotherapy

In many chemotherapeutic drugs, nephrotoxicity due to dose limitation is a serious concern in oncology. Although the pathophysiology of chemotherapy-induced AKI is diverse, the emergence of severe mitochondrial dysfunction is a basic element across different drug classes. The mitochondria is the main target organelle in nephrotoxicity in the renal proximal tubule due to the large number of mitochondria and dependency on oxidative metabolism [52]. For instance, one-third of patients suffer from nephrotoxicity after using cisplatin, which is actively taken up by renal tubular cells. There, it builds a concentration greater than the plasma. It slows down the oxidation of fatty acids, disturbs the electron transport chain (ETC), and causes oxidative stress in mitochondria [15,53,54]. Specifically, cisplatin impairs ETC complexes I, II, and IV, collapsing the proton gradient and halting ATP-synthase (Complex V) activity [55].

At the same time, the electron leak produces superoxides and causes a sharp increase in ROS [56]. Mitochondrial injury in AKI involves multiple interconnected mechanisms. The oxidative assault triggers mitochondrial outer membrane permeabilization and the opening of the mitochondrial permeability transition pore (mPTP), releasing cytochrome c and activating caspase-9 and downstream caspases, ultimately driving tubular cell death. An important protective mechanism is mitophagy, which removes damaged mitochondria via autophagy. Mitophagy attempts to limit cisplatin-induced AKI, but it is often quickly overwhelmed. Dysregulation of key regulators such as PINK1 causes injured mitochondria to become a persistent source of reactive oxygen species (ROS), further amplifying cellular injury [57].

Several pharmacological agents exacerbate mitochondrial dysfunction. Methotrexate (MTX), often combined with cisplatin, reduces tubular bioenergetics: in vitro and in vivo studies show that MTX decreases ATP levels, increases ROS, and inhibits mitochondrial respiratory chain activity [58,59]. Clinically, a high-dose of MTX is associated with AKI incidence ranging from 1.8% to 9.1% [57,60,61]. Tyrosine kinase inhibitors (TKIs), such as sunitinib and sorafenib, similarly disrupt mitochondrial function by inhibiting complexes I and III, impairing ATP production, and inducing ROS-driven apoptosis [62]. Recent mechanistic studies have added translational significance; for example, the activation of PGC-1α mitigated AKI induced by cisplatin by restoring mitochondrial biogenesis, whereas TRPM2 channel clearing increased mitochondrial damage in models treated with cisplatin [62,63]. Though there is a change in the chemical composition and target of the various anticancer drugs, they all have a common pathway of mitochondrial damage [64]. This causes a bioenergetic crisis from ATP depletion, and a lack of cellular energy impairs the transcription of proteins required for mitochondrial repair [45]. These advances suggest that mitochondrial protection is important to address nephrotoxicity. Off-target effects are another concern in multi-treatment oncology. Future studies should therefore incorporate prospective designs, mitochondrial endpoint assessments, and integrate oncologic and renal outcomes.

## 6. Acute Kidney Injury and Mitochondrial Dysfunction During Immune Checkpoint Inhibitor Therapy

Immune checkpoint inhibitors (ICIs) have transformed oncology, offering a new therapeutic approach for some patients with metastatic cancers. However, by activating T-cell-mediated immune responses against tumors, these drugs can also trigger a range of immune-related adverse events (irAEs). Acute kidney injury (AKI) has emerged as one of the notable complications. Its pathophysiology is distinct, often driven by acute interstitial nephritis (AIN) [65]. Several studies on this topic are summarized in Table 1.

Epidemiological data from real-world cohorts indicate that clinically significant AKI following ICI therapy is not uncommon. A large retrospective study reported that over 20% of patients developed some form of AKI, with roughly 10% experiencing sustained, severe (stage 2 or 3) AKI within 12 months of starting treatment [73]. Among these severe cases, about one-third were attributed to potential ICI-related toxicity. This highlights the importance of careful evaluation to distinguish immune-mediated injury from other common causes such as prerenal azotemia, sepsis, or nephrotoxicity from concomitant medications. The hallmark of immune checkpoint inhibitor (ICI)-associated AKI is a T-cell-driven immune response. Renal biopsies consistently reveal dense tubulointerstitial infiltrates predominantly composed of CD3^+^ T cells, with minimal involvement of B cells, plasma cells, or eosinophils [66]. This histopathological pattern is consistent with ICI-related toxicity resulting from the loss of peripheral immune tolerance.

Current evidence suggests that ICIs disrupt regulatory T-cell-mediated immune control by blocking the co-inhibitory receptors PD-1 and CTLA-4. This dysregulation may either lead to a de novo autoimmune response against renal antigens or, more intriguingly, to the reactivation of memory T cells previously primed in the presence of exogenous haptens [74]. Clinical data further support this model, as many patients with ICI-associated AIN had concurrent exposure to drugs known to induce AIN, such as PPIs and NSAIDs [65]. This suggests that ICIs may lower immune tolerance thresholds, enabling the activation of pre-existing drug-specific T-cell responses. While ICIs enhance anti-tumor immunity by blocking CTLA-4 and PD-1/PD-L1 signaling, this immune activation may also result in renal off-target toxicity, most frequently presenting as acute tubulointerstitial nephritis with lymphocytic and monocytic tubular infiltration [75]. The mitochondria are central in ICI-related AKI (ICI-AKI): they are the focus of hyper-energized T cells that damage renal tissue [76]. Hyperactivated T cells convert the metabolic program from mitochondria-driven oxidative phosphorylation (OXPHOS) to aerobic glycolysis, which allows for the rapid proliferation and accumulation of ROS and damage to the mitochondrial DNA [63]. Kidney biopsy is the most recommended method of diagnosing AIN and is highly recommended where the diagnosis is not clear or the presentation is unusual. Most patients respond positively to steroids, and a high percentage of them recover renal function, emphasizing the reversibility of the injury when early intervention is performed [73].

Overall, AKI is a serious immune-related adverse event of ICI therapy, primarily driven by T-cell-mediated AIN. Timely diagnosis requires high clinical suspicion, the careful exclusion of other causes, and awareness of concomitant medications [77] that may contribute to injury. As the use of ICIs grows, prospective studies with systematic biopsy protocols are needed to define precise antigenic targets and to develop biomarkers that can predict and diagnose ICI-AKI more accurately. Clinically, ICI-AKI occurs in approximately 2–5% of treated patients, with ATIN as the predominant pathology. A meta-analysis found a pooled incidence of 1.4% (95% CI 1.0–2.1%) and an 18% recurrence rate upon ICI rechallenge [73]. A history of PPI or NSAID use and existing kidney disease are some of the risk factors. Regardless of these observations, there are still several gaps. The number of studies that examined mitochondrial biomarkers in ICI-AKI is very small, most of the evidence is based on observations, and because of the heterogeneity of the types of ICI, doses, and comorbid conditions of patients, the generalizability is limited. The potential trials to be performed to protect mitochondria remain.

## 7. Mitochondrial Dysfunction and Acute Kidney Injury Following CAR T-Cell Therapy

Acute kidney injury (AKI) is a relatively common complication following CAR T-cell therapy, occurring in up to 20% of patients within the first 30 days post-infusion, with severe cases (stage 2–3) being less frequent and there being the occasional need for kidney replacement therapy [78]. AKI typically develops within the first two weeks, often during the initial week, and about half of affected patients recover baseline renal function within 30 days. Its development is closely associated with cytokine release syndrome (CRS) and neurotoxicity, highlighting a likely immune-mediated mechanism. In particular, the massive release of pro-inflammatory cytokines, including IL-6, IL-1β, IFN-γ, and GM-CSF, by infused CAR T cells and activated host immune cells drives a systemic hyperinflammatory state that may induce mitochondrial injury in renal tubular cells [78]. This mechanism may lead to impaired oxidative phosphorylation, increased mitochondrial reactive oxygen species (mtROS), and the activation of cell death pathways, which together contribute to AKI. Traditional pre-treatment risk factors have shown limited predictive value, though additional contributors include prior chronic kidney disease, exposure to antibiotics, and intravenous contrast. In most cases, AKI is transient and reversible, but severe episodes can have significant clinical consequences. Despite increasing recognition, the precise pathophysiology remains incompletely understood, and long-term outcomes, particularly in patients with pre-existing CKD, are poorly characterized. These observations underscore the need for multicenter prospective studies to better define mechanisms, including inflammation-driven mitochondrial damage, identify high-risk patients, and develop preventive and therapeutic strategies to mitigate CAR T-cell-associated kidney injury [79,80].

## 8. Emerging Biomarkers of Mitochondrial Distress

The detection of mitochondrial dysfunction as a central-playing process has facilitated the exploration of biomarkers that can be used to recognize renal injury before it is reflected by an increase in serum creatinine. These biomarkers that are produced by mitochondria provide an opportunity for early diagnosis. One of the top candidates is cell-free mitochondrial DNA (ccf-DNA), which is discharged when cells are harmed. As a DAMP, ccf-mtDNA triggers an innate immune reaction and inflammation. It has clinical significance, as high concentrations are seen in patients following cardiac surgery, in which it is associated with the severity and duration of AKI [81,82]. Other possible biomarkers are proteins indicating mitochondrial dynamics (e.g., Drp1) or mitophagy (e.g., PINK1) in the urine or trace metabolic products in the blood or urine (e.g., high levels of succinate, acylcarnitine) that suggest changes in energy metabolism and failure of mitochondrial oxidation. The discovery of such biomarkers would allow clinicians to be able to detect patients at high risk and put up protective measures during the critical period before they suffer irreparable harm [83]. The critical role of mitochondrial dysfunction in AKI, has radically changed the paradigm of their role and treatment. The mitochondria become from downstream effector the direct regulator and a cause of the catastrophe into the cells. Additionally the novel strategic treatment goals include breaking the vicious cycle of injury at its source, by maintaining mitochondrial integrity, strengthening it, and favoring its rehabilitation. It is a great improvement over the past interventions that attempted to inhibit isolated inflammatory or cell death pathways once they had become fully activated. These therapies are developed with our precise knowledge of the biology of the mitochondrion and are divided in terms of the modulated pathological process [84].

**Figure 4:**
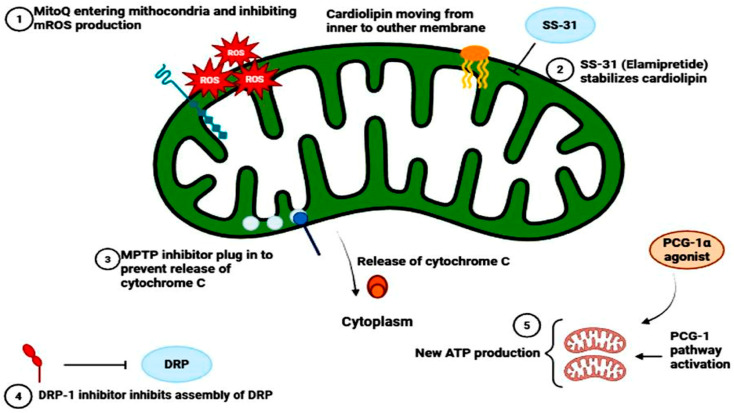
Multi-faceted therapeutic strategy to preserve mitochondrial integrity in AKI. (**1**) Mitochondria-targeted antioxidants (MitoQ) scavenge ROS and protect mitochondria. (**2**) SS-31 stabilize cardiolipins to stabilize mitochondrial membrane. (**3**) Cell death inhibitors block the mPTP to prevent the point of no return in apoptosis. (**4**) PGC-1α agonists promote the renewal and clearance of mitochondria. (**5**) Dynamic modulators inhibit pathological fission to maintain a functional network.

The former and the best studied category is that of mitochondria-targeted antioxidants. The breakdown of the traditional, non-targeted antioxidants in a clinical trial on AKI can be explained by the fact that they cannot accumulate in the mitochondrial matrix, which is the major location of ROS generation. This has been circumvented by conjugating the antioxidant moieties with lipophilic cations such as tripheny Iphoshonium (TPP+), which enables their entry into the lipid bilayers, as well as their concentration in the negatively charged mitochondrial matrix by several hundred-fold [85]. The most successful of these include MitoQ (ubiquinone conjugated to (TPP+) and Mito TEMPO (a superoxide dismutase mimetic conjugated to TPP+), which have shown excellent efficacy in pre-clinical models [86]. These compounds have a powerful inhibitory effect on oxidative lipid damage, protein and DNA damage, ameliorate mitochondria bioenergetics, mitigate inflammation, and eventually maintain renal function by scavenging ROS at its source in environments of ischemia–reperfusion and cisplatin-induced nephrotoxicity [26]. SS-31 (Elamipretide), a small peptide that binds the inner membrane of the mitochondrion, is another promising Mito protective agent. It interacts with cardiolipin, which is a phospholipid that is vital in the architecture and functionality of ETC super complexes. SS-31 stabilizes cardiolipin, inhibiting its peroxidation, improving the efficiency of electron transfer, reducing ROS leakage and mPTP opening, and has strong protective effects in a wide range of AKI models [87]. The second strategic direction is mitochondrial dynamics modulation. Since the pathological changes are the shift towards over fission through Drp 1 in AKI, it is an evident target of therapy to curb this process [52]. Mdivi-1 and other pharmacological Drp 1 inhibitors have great potential in animal models. Mdivi-1 inhibits Drp1 GTPase activity, leading to the pathological fragmentation of the mitochondrial network, which is useful in preserving a more fused functional state. The outcomes of treatment include the inhibition of ROS generation, a decrease in cytochrome c release, a decrease in the levels of apoptosis, and a complementary approach to improve mitochondrial complementation and resilience [88]. Efforts are underway to develop pharmacological agents that activate or stabilize the fusion proteins mitofusin (Mfn 1/2) and OPA1, though this approach is in the earlier stages of development [89]. (Figure 4).

## 9. Therapeutic Strategies Targeting Mitochondria

Rather than merely preventing damage, a third strategy focuses on boosting the kidney’s innate repair mechanisms by enhancing biogenesis and quality control. Promoting the generation of new, healthy mitochondria is achieved by targeting PGC-1α, the master regulator of biogenesis. As Table 2, compounds like Resveratrol and more specific PGC-1α agonists (e.g., SR-18292) have been shown to upregulate mitochondrial mass, improve oxidative capacity, and accelerate functional recovery in AKI models [24,90]. Simultaneously, enhancing the clearance of damaged organelles is critical for quality control. The mTOR inhibitor rapamycin and newer, more specific mitophagy inducers like Urolithin A (a natural metabolite that promotes mitophagy) have demonstrated protective effects by accelerating the removal of dysfunctional mitochondria, reducing apoptosis, and improving tubular regeneration post-injury [91,92]. Finally, inhibiting the point of no return, the execution of cell death is a key goal. Cyclosporine A is a well-known inhibitor of the mitochondrial permeability transition pore (mPTP) opening. While its nephrotoxic side effects preclude chronic use, its administration at the time of reperfusion has shown protective effects in the models of renal IRI, underscoring the therapeutic value of mPTP inhibition [93]. The development of more specific mPTP inhibitors without immunosuppressive effects is an active area of research. Additionally, agents that inhibit the activation of Bax/Bak or broad-spectrum caspase inhibitors could theoretically prevent the execution phase of apoptosis, preserving tubular cells, though their clinical translation has been challenging [91,92].

Several clinical trials are currently investigating the mechanisms and prevention of acute kidney injury (AKI) in cancer patients receiving chemotherapy or immunotherapy, with a particular focus on the potential role of mitochondrial protection. For example, NCT07018622 is a Phase II study evaluating the nephroprotective effect of dapagliflozin in patients receiving cisplatin for solid tumors, with endpoints including renal function recovery, AKI severity, and safety. Similarly, NCT05640817 explores whether pentoxifylline can reduce oxidative stress and inflammation induced by cisplatin, assessing renal recovery and mitochondrial biomarkers in a Phase I/II design. NCT01848457, a completed Phase II trial, examined the use of the proton pump inhibitor pantoprazole during high-dose methotrexate therapy in osteosarcoma patients, measuring renal outcomes and inflammatory markers. On the immunotherapy side, NCT07101913 is an ongoing observational study investigating the incidence and severity of AKI after CD19 CAR T-cell infusion in B-cell lymphoma, including patients with baseline chronic kidney disease, and evaluating CRS-related renal outcomes. Finally, NCT06549634 is assessing biomarkers of AKI in multiple myeloma patients receiving daratumumab, focusing on the incidence and severity of renal injury. Collectively, these trials highlight two complementary approaches: targeting mitochondrial injury with antioxidant or mitochondrial-protective strategies and systematically monitoring renal toxicity during immunotherapy. Such studies may clarify whether mitochondrial preservation can prevent AKI and to what extent immunotherapy itself contributes to renal injury in cancer patients. Overall, available clinical data suggest that targeting immune–mitochondrial pathways holds clinical promise, but they also emphasize the need for adequately powered, mechanistically informed trials to translate biological rationale into meaningful renal outcomes (Table 3).

## 10. Future Prospectives

Although there has been a substantial development in the field of comprehending the role of mitochondrial dysfunction in AKI, there is still an effort to adapt these findings into viable functional clinical treatment. The future of this research area will be based on the development of the gap between discovery and therapeutic applications. Surpassing examples of metabolomics, transcriptomics, and single-cell proteomics, multi-omics will provide a new repertoire of mitochondrial injury in AKI in the wake of different etiologies, such as ischemia–reperfusion, chemotherapy, and immunotherapy-induced injury. A combination of the information provided by these with artificial intelligence (AI)-based predictive models may allow us to diagnose and provide personalized treatment early. The recent technological advances, including organoids, 3D bio-printed nephron systems, and microfluidic organ-on-chip models, are potent in coping with the goal of recapitulating human AKI pathophysiology in mitochondrial detail. These models have the potential to transform drug testing, where high-throughput screening of mitochondria-targeted compounds has been performed using a better translation approach. Further studies of mitochondrial transfer using extracellular vesicles and stem cell-mediated mitochondrial therapy might provide a new frontier in renal regeneration. To develop future generation therapeutics aimed at altering energy metabolism and inflammation simultaneously, a better understanding of the cross-talk between immune cells and tubular epithelium and how it is regulated by immune-metabolomic reprogramming and mitochondrial communication will be essential. Mitochondrial modulators can be used along with oncology and cardiotherapy to provide protection against Aki without affecting the anticancer activity, which calls for extensive pharmacological optimization and validation.

## 11. Conclusions

Mitochondrial dysfunction represents a common crossroads in AKI caused by ischemia–reperfusion injury, chemotherapy, and immunotherapy. In chemotherapy-associated AKI, mitochondrial damage is mainly driven by direct toxicity, including injury to mitochondrial DNA, impaired oxidative phosphorylation, and defective mitophagy. In contrast, immunotherapy-related AKI is largely mediated by immune activation, cytokine excess, and inflammation-induced metabolic stress, leading to secondary mitochondrial dysfunction. Beyond energy production, mitochondria act as central regulators of cell fate, immunity, and inter-organ communication. Disruption of mitochondrial bioenergetics and quality control promotes oxidative stress and inflammation, favoring the transition from AKI to chronic kidney disease.

Therapeutic strategies targeting mitochondrial integrity show promising renoprotective effects in experimental models. However, their clinical translation faces substantial challenges. Most mitochondrial-targeted interventions lack tissue specificity and may protect tumor cells by enhancing mitochondrial fitness. This risk is particularly relevant in patients receiving chemotherapy or immune checkpoint inhibitors, where mitochondrial function directly influences treatment efficacy. Moreover, modulation of immune–mitochondrial pathways may blunt anti-tumor immune responses or exacerbate immune-related adverse events. Safety concerns are amplified in combination regimens, where overlapping toxicities, drug–drug interactions, and cumulative mitochondrial stress are common. In addition, current evidence is largely derived from preclinical studies, with limited validation in cancer-relevant AKI models. The absence of validated mitochondrial biomarkers further limits patient stratification and treatment monitoring. These limitations underscore the need for early, kidney-targeted interventions, precision medicine strategies, and mitochondria-focused clinical trial designs that explicitly incorporate oncological outcomes. Only through the integration of mitochondrial biology, immunology, and regenerative medicine can nephroprotection be achieved without compromising cancer control.

## Figures and Tables

**Table 1:** Mechanistic, preclinical, and clinical evidence linking immune checkpoint inhibitors to acute kidney injury (AKI).

Aspect	Preclinical Evidence	Clinical Evidence	Limitations/Comments	References
T-cell metabolic reprogramming	Hyper-activated T cells switch from OXPHOS to aerobic glycolysis; ROS accumulation damages mtDNA	Human biopsies profile lymphocytes’ inflammatory infiltrates	Translational gap; need mitochondrial biomarkers in patient samples	[66]
Cytokine-mediated tubular injury	IFN-γ, TNF-α impairs ETC, increases ROS, induces Drp1-mediated fission, and impairs PINK1-Parkin mitophagy in tubular cells	Urinacytokines Kidney biopsies in ICI-AKI show ATIN and mitochondrial swelling	Small sample size; mostly case reports or single-center studies	[67,68]
Mitochondrial ROS and oxidative stress	ROS causes ETC dysfunction, mtDNA damage, and bioenergetic collapse, amplifying inflammation	Clinical data limited; indirect evidence from systemic oxidative stress markers	Lack of longitudinal ROS measurements in patients; heterogeneous regimens	[69]
Immune activation amplification	Upregulation of MHC, ICAM-1, and adhesion molecules enhances T-cell adhesion and tubular inflammation	Clinical correlation: histology shows increased lymphocyte infiltration and tubular injury	Mostly observational; mechanistic causality not confirmed	[70]
Incidence and risk factors	NA	ICI-AKI incidence 2–5%; risk factors: CKD, PPI/NSAID use, and ICI combination therapy	Limited prospective data; small cohorts, mostly retrospective	[68,71,72].

**Table 2:** Clinical perspectives and translational readiness of mitochondria-targeted therapies in AKI.

Therapeutic Strategy	Mechanistic Rationale	Clinical Application	Current Clinical Evidence	Translational Limitations	References
Mitochondria-targeted antioxidants	Scavenge mtROS at the sources; stabilize respiration	IRI patients (cardiac patients), cisplatin therapy, and sepsis AKI	Small clinical studies show reduced oxidative biomarkers but limited AKI-specific trials	Optimal dosing unknown, no validated mitochondrial biomarkers; heterogenous patient cohorts	[52,54]
SS-31 (Elamipretide)	Stabilize cardiolipin and ETC super-complexes	High-risk surgical patients; chemotherapy recipients	Pilot studies report improved renal perfusion and reduced injury markers	Large RCTs lacking; cost and route of administration limit use	[87]
PGC-1α activator (Resveratrol SR-18292)	Enhances biogenesis and ATP renewal	Chemotherapy-AKI, ICI-AKI, metabolic AKI	Preclinical evidence is strong; no Phase II/III trials	Poor Pharmokinetics; unclear effect on critically ill patients	[24,90]
Drp1 inhibitor (Mdivi-1)	Block excessive fission, reduce apoptosis	Ischemic AKI, cisplatin nephrotoxicity	No human trials yet; strong preclinical nephroprotection	Concern regarding off-target effects and long-term safety	[11,88]
Mitophagy enhancers (Cyclosporin)	Clear damaged mitochondria; reduce inflammation	Post-IRI AKI, sepsis AKI	Early human safety data for Cyclosporin; no AKI-specific trials	Timing critical; risk of immunosuppression (rapamycin)	[33,93]
pathway inhibitors (TLR9 blockers, STING inhibitors, and NLRP3 inhibitors)	Reduced inflammation triggered by mt DNA, cardiolipin	ICI-AKI, CAR-T AKI, and ischemic AKI	Human-based data minimal; early-phase testing ongoing in inflammatory disease	Risk of impairing host defense; limited kidney-specific studies	[26,36,40]
Mitochondrial transfer/Stem-cell mitochondrial therapy	Replace damaged mitochondria; restore energetics	Pediatric AKI, chemotherapy nephrotoxicity	Limited to animal models	Ethical and technical challenges	[82,94]

**Table 3:** Completed and ongoing clinical trials in cancer patients with acute kidney injury (AKI) receiving chemotherapy or immunotherapy.

Clinical Trial/Identifier	Condition/Population	Intervention/Target	Condition	Study Design/Phase	Status	Main Outcomes/Endpoints
NCT07018622	Protecting the Kidney Proximal Tubules From Platinum-Based Chemotherapy Toxicity	Drug: Dapagliflozin Drug: Placebo	Solid tumors Cisplatin nephrotoxicity	Phase II	Ongoing	Renal function recovery, AKI severity, and safety
NCT07101913	Occurrence of Acute Kidney Injury After CAR T-Cell Treatments in B-Cell Lymphoma	Biological: Incidence of acute kidney injury Describe risk factors in AKI occurrence Other: Focus on patients with chronic kidney disease at baseline	Acute kidney injury (AKI) Infusion of CD19 CAR T cell B-cell lymphoma	Observational	Ongoing	Incidence and severity of AKI, CRS-related renal outcomes
NCT06549634	Biomarkers of AKI in Patients Receiving Daratumumab	Drug: Daratumumab	Acute kidney injury Multiple myeloma Light-chain nephropathy	Observational	Ongoing	Incidence and severity of AKI
NCT05640817	Nephroprotective Effect of Pentoxifylline Against Cisplatin in Patients with Head and Neck Cancer	Drug: Pentoxifylline 400 mg SR tablets Drug: Cisplatin with standard hydration with normal saline	Pentoxifylline, reduced the oxidative stress and inflammation induced by cisplatin	Phase I/II	Unknown status	Safety, mitochondrial biomarkers, renal recovery
NCT01848457	Preventing Nephrotoxicity and Ototoxicity from Osteosarcoma Therapy	Drug: Pantoprazole mitochondrial pump inhibitor Drug: High-dose methotrexate infusion duration	Protonic pump inhibitor	Phase II	Completed	Renal outcomes, inflammatory marker reduction

## Data Availability

The original contributions presented in this study are included in the article. Further inquiries can be directed to the corresponding author(s).

## Abbreviations

The following abbreviations are used in this manuscript:

AIN	Acute Interstitial Nephritis
ATIN	Acute Tubulointerstitial Nephritis
ATN	Acute Tubular Necrosis
CKD	Chronic Kidney Disease
CRS	Cardio Renal Syndrome
DRP1	Dynamin Related Protein 1
DAMP	Damage Associated Molecular Pattern
ETC	Electron Transport Chain
GFR	Glomerular Filtration Rate
ICI	Immune Checkpoint Inhibitor
irAE	Immune Related Adverse Event
KDIGO	Kidney Disease: Improving Global Outcomes
MAVS	Mitochondrial Antiviral Signaling Protein
MHC	Major Histocompatibility Complex
mtROS	Mitochondrial Reactive Oxygen Species
NGAL	Neutrophil Gelatinase-Associated Lipocalin
NSAID	Non-Steroidal Anti-Inflammatory Drug
PINK1	PTEN-Induced Kinase 1
